# A Codesigned Approach to Develop Tailored Antimicrobial Stewardship Strategies in Bangladesh: A Social–Ecological Systems Framework Analysis

**DOI:** 10.1111/hex.70822

**Published:** 2026-08-17

**Authors:** Abdullah Al Masud, Kamal Ibne Amin Chowdhury, Ramesh Lahiru Walpola, S. M. Zafar Shafique, Tamanna Sultana, Maria Akter, Nisharggo Niloy, Md Saiful Islam, Holly Seale

**Affiliations:** ^1^ School of Population Health Faculty of Medicine and Health University of New South Wales Sydney Australia; ^2^ International Centre for Diarrhoeal Disease Research, Bangladesh (ICDDR,B) Dhaka Bangladesh; ^3^ School of Health Sciences, Faculty of Medicine and Health University of New South Wales Sydney Australia

**Keywords:** antimicrobial resistance, antimicrobial stewardship, codesign research, community‐pharmacies, inappropriate antibiotic use, over‐the‐counter antibiotic sales, social–ecological systems framework

## Abstract

**Background:**

Multistakeholder collaboration and codesign are increasingly recognized as effective approaches for addressing complex public health challenges such as antimicrobial resistance (AMR), yet stewardship efforts in low‐ and middle‐income countries remain fragmented and are often limited to education‐focused interventions with minimal community engagement. This study used a codesign approach to develop context‐specific antimicrobial stewardship (AMS) strategies for community pharmacies in rural Bangladesh, where over‐the‐counter antibiotic sales are common and pharmacy‐led care is limited.

**Methods:**

Separate codesign workshops were conducted in two rural regions of Bangladesh with stakeholders involved in antibiotic marketing, prescribing, dispensing, and use. Guided by the Social Ecological Systems Framework, participants identified priorities and feasible interventions at the systemic, organizational, community, and individual levels.

**Results:**

Stakeholders proposed a comprehensive, interconnected package of strategies spanning all system levels to strengthen AMS and promote appropriate antibiotic use. System‐level priorities included improving access to public healthcare, strengthening diagnostic and referral systems, promoting guideline adherence, clarifying informal providers' roles, engaging pharmacy associations, and strengthening regulation of antibiotic sales and unlicensed practice, though constrained by limited resources, political inertia, conflicts of interest, and policy gaps. At the organizational level, recommendations included antibiotic‐specific packaging and labelling, physician counselling on treatment completion, and reinforcement of stewardship messages by drug‐sellers, though implementation may be limited by commercial interests, workforce shortages, affordability concerns, and drug‐sellers' limited AMR knowledge. At the community level, stakeholders emphasized public awareness campaigns, school‐based education, and engagement of nongovernmental and community organizations, noting that awareness alone is unlikely to succeed without enforcement. At the individual level, patients were encouraged to seek advice on appropriate antibiotic use, though participants noted that sustained behaviour change would require broader system reforms, and that isolated interventions were unlikely to succeed without coordinated action across multiple health system levels.

**Conclusions:**

Effective AMR mitigation requires coordinated, multisectoral action integrating policy reform, regulatory enforcement, community engagement, and contextually appropriate stewardship interventions. These findings highlight the value of codesign in identifying feasible strategies that address both structural and behavioural drivers of antibiotic use, considering the local social and economic realities.

**Patient or Public Contribution:**

Patients, community members, community‐pharmacy drug‐sellers, pharmaceutical sales representatives, and registered physicians from both public and private facilities, along with local health authorities, actively contributed to this study through workshops. They shared lived experiences from their respective roles and provided contextual insights that informed the design and interpretation of the findings. Their involvement ensured that the stewardship strategies were relevant and appropriate for rural community‐pharmacy settings in Bangladesh.

**Abbreviations**
ABACUS: AntiBiotic ACcess and USe
AMR: antimicrobial resistance
AMS: antimicrobial stewardship
BMDC: Bangladesh Medical and Dental Council
DGDA: Directorate General of Drug Administration
GARDP: Global Antibiotic Research and Development Partnership
MBBS: Bachelor of Medicine, Bachelor of Surgery
NAP: National Action Plan
NGOs: nongovernment organizations
NRAs: National Regulatory Authorities
OTC: over‐the‐counter
SACMO: Sub‐Assistant Community Medical Officer
SESF: social–ecological systems framework
STGs: standard treatment guidelines

## 1 Introduction

Antimicrobial resistance (AMR) is a major global health threat, with bacterial AMR‐attributable deaths projected to increase from 1.14 million in 2021 to 1.91 million by 2050 and AMR‐associated deaths expected to reach 8.22 million annually, with the greatest burden projected in low‐ and middle‐income countries, particularly in South Asia [1]. Bangladesh, a densely populated South Asian low‐ and middle‐income country experiencing rapidly increasing AMR, faces substantial public health challenges due to the widespread availability and inappropriate use of antibiotics [2, 3]. Recent research in Bangladesh shows that community pharmacies dispense 56.6% of antibiotics without prescriptions [4]. This issue is complex and stems from social, economic, healthcare, manufacturing, supply chain, and policy factors, requiring solutions beyond awareness and healthcare improvements to include sustainable manufacturing, economic considerations, and stakeholder empowerment [5]. Effective strategies to address these issues require an understanding of provider behaviours, service and economic priorities, and consumer needs to help reduce antibiotic resistance [6]. Different stakeholders, such as pharmaceutical companies and healthcare providers, often have conflicting interests that can impede the effective implementation of AMR policies [7].

AMR is not merely a biological issue but is deeply linked to health systems, social behaviours, economic contexts, and social inequalities in LMICs, where factors like poverty, limited healthcare access, and poor living conditions drive resistance, highlighting the need for integrated, multilevel strategies that address social determinants alongside biomedical interventions [8, 9]. Bringing together stakeholders from different disciplines is highly beneficial for problem exploration, such as the challenge of AMR [10]. To mitigate these challenges, codesign processes and practices are increasingly being used to support collaborative exploration to devise solutions [11]. Several reviews noted that antimicrobial stewardship (AMS) in LMICs is often fragmented, with limited community‐based research—typically education‐focused and single‐component—and that community engagement or codesign methods are underused, highlighting the need for more multifaceted, context‐specific, and stakeholder‐engaged approaches [12, 13]. The WHO‐Bangladesh policy guidance highlights stakeholder‐engaged regulatory action, showing that policy shifts benefit from multistakeholder data and engagement, while underscoring the need for but scarcity of broad codesigned strategies in LMICs [14].

This study aimed to develop a comprehensive, context‐specific AMS strategy by engaging key stakeholders involved in antibiotic marketing, prescribing, dispensing, and use in nonhospital and community settings, particularly community pharmacies. Guided by the Social Ecological Systems Framework, the study sought to generate contextually relevant and actionable AMS strategies that are feasible within local resource constraints and capable of informing future policy, programme development, and implementation efforts across the health system, organizational, community, and individual levels.

## 2 Methods

### 2.1 Study Settings

**Photo 1:**
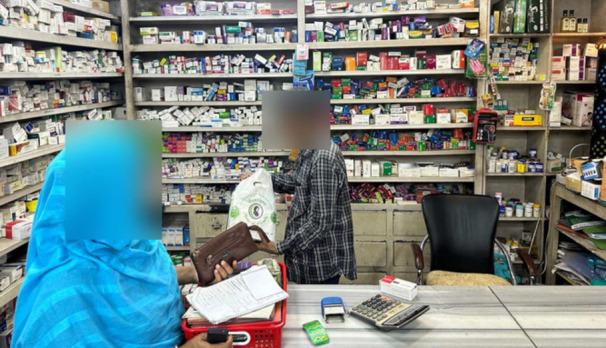
A typical community pharmacy in Bangladesh.

**Photo 2:**
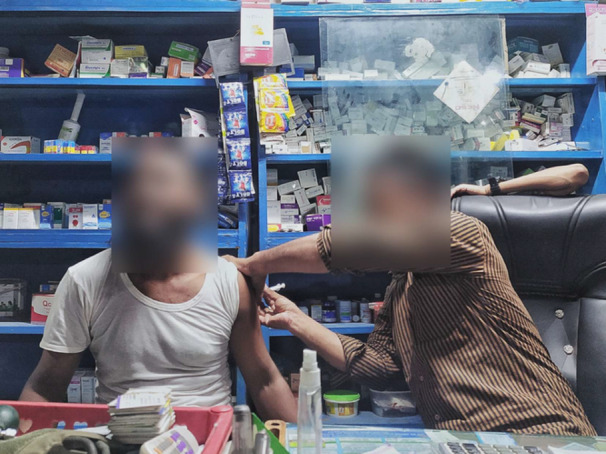
Pharmacy drug‐sellers serving as “village doctors.”

Healthcare in Bangladesh is delivered through a pluralistic system comprising public facilities, private hospitals and clinics, village doctors, and community pharmacies operating within a loosely regulated environment [15]. Persistent shortages and uneven distribution of formally trained professionals, particularly in rural areas, have sustained reliance on informal providers as accessible, affordable care sources [16]. The formal sector consists of physicians holding a Bachelor of Medicine, Bachelor of Surgery (MBBS) degree recognized by the Bangladesh Medical and Dental Council (BMDC), whereas informal village doctors lack formal qualifications yet remain trusted for their longstanding community presence [17, 18]. These providers often serve as the first point of contact but operate with limited regulatory oversight and inappropriate antibiotic use [16]. Community pharmacies are also a major first point of care, with over 80% of the population seeking treatment from drug‐sellers [19, 20]. A scoping review identified 117,354 registered retail pharmacies nationwide, approximately 7.2 per 10,000 population [21]. Pharmacies require licensing from the Directorate General of Drug Administration (DGDA), which sets standards for infrastructure, storage, and staff qualifications [21, 22, 23], and national policy prohibits dispensing antibiotics without a prescription [24, 25]. Weak enforcement has led to widespread noncompliance, including routine nonprescription antibiotic sales [23, 26, 27]. Drug‐sellers mainly dispense medicines, whereas village doctors also consult, advise, and prescribe despite lacking formal training, with roles frequently overlapping [22, 28]. Both groups are often grouped as informal or unqualified allopathic providers [29], and recent studies show these boundaries are increasingly fluid [30, 31]. Many pharmacies lack trained pharmacists and are staffed instead by drug‐sellers, a distinction that matters because pharmacists provide counselling and medication management while sellers focus mainly on sales, with implications for antibiotic stewardship [23, 26, 27]. Antibiotic use is slightly higher in rural areas, where nonprescription purchases are more common, and treatment adherence is 37% lower than in urban areas [4, 32], reflecting greater rural reliance on informal providers, consistent with earlier research [33, 34, 35] (Photos 1, 1 and 2, 2).

Building on these findings, the present study was conducted in two rural subdistricts located in northern and southwestern Bangladesh. These settings are characterized by limited access to formal healthcare services, a high reliance on informal healthcare providers, and the prominent role of community pharmacies in primary healthcare delivery.

### 2.2 Workshop Participants

A purposive stakeholder‐based sampling strategy was used to recruit participants for codesign workshops conducted in two rural areas of Bangladesh. Four stakeholder groups were included: community antibiotic users, drug‐sellers, registered physicians, and pharmaceutical sales representatives. Community antibiotic users aged 18 years or older who had recently purchased antibiotics for themselves or a family member and resided in the local community were identified and approached directly by trained members of the research team outside pharmacies and invited to attend workshops scheduled in the following weeks. Drug‐sellers included pharmacy owners and unregistered village doctors involved in dispensing antibiotics, with at least 1 year of relevant experience. Registered physicians and pharmaceutical sales representatives were identified through local professional networks and healthcare facilities and were approached directly by the research team. Individuals who declined participation or were unavailable were not pursued further, and no identifying information was retained. To enhance diversity of perspectives, participants were purposively recruited across different professional roles, practice settings, and communities within the two study sites. None of the participants had an established research relationship with the study team before recruitment.

Lists of pharmacies were obtained from local Chemists and Druggists Association/Samity, the subdistrict‐level branch of the Bangladesh Chemists and Druggists Samity, operating under the respective District Chemists and Druggists Association/Samity. Pharmacies were purposively selected from the list to ensure variation in service type and location, including small medicine dispensing outlets, larger pharmacies offering physician consultations or operated by locally recognized village doctors, pharmacies located in village markets or major transport hubs, community‐based pharmacies, and Model Medicine Shops. Registered physicians were identified using lists obtained from local civil surgeons' offices and were required to hold an MBBS degree, be registered with the BMDC, and actively practice in public or private healthcare facilities. Pharmaceutical sales representatives were required to have at least 1 year of experience in antibiotic marketing. They were identified through nominations provided by drug‐sellers, who listed antibiotic‐supplying companies with a strong local presence. Representatives from the most frequently nominated companies were subsequently approached for participation.

As this was a codesign study, participant numbers were determined pragmatically to ensure representation of diverse stakeholder perspectives and effective group discussion, rather than to achieve statistical power. For community antibiotic users, drug‐sellers, and pharmaceutical sales representatives, a target of 20–24 participants per workshop was established to facilitate division of each stakeholder group into two subgroups of approximately 10–12 participants. To achieve this target, an additional 10–15 participants from each stakeholder group were invited in each study area. For registered physicians, a single group of 10–12 participants was planned for each workshop because of the limited number of physicians available in each upazila and their professional commitments. Therefore, 15 to 18 physicians were invited in each area to minimize the impact of nonparticipation. All interested participants received a recruitment leaflet describing the study objectives and procedures. The final sample comprised 39 community antibiotic users, 37 drug‐sellers, 47 pharmaceutical sales representatives, and 12 registered physicians across the two study sites. Participants received a modest reimbursement in accordance with institutional guidelines to compensate for their time, travel, and any associated expenses incurred through participation in the workshops.

### 2.3 Codesign Sessions

A sequential, codesign approach was adopted, comprising separate workshops with antibiotic users, drug‐sellers, pharmaceutical sales representatives, and registered physicians. Workshops were conducted separately to account for differences in professional roles, educational backgrounds, and potential conflicts of interest among stakeholder groups, as well as the practical constraints of convening all participants simultaneously. Although held separately, the workshops were not conducted in isolation. Key findings from each session were systematically synthesized and carried forward to inform subsequent workshops, enabling participants to engage with and respond to the perspectives of preceding stakeholder groups. The workshop with antibiotic users was conducted first, followed by drug‐sellers, to whom relevant insights from the users' session were presented for consideration. Building on this, the pharmaceutical sales representatives' workshop incorporated key findings from both preceding groups, offering insight into the practical realities faced by users and drug‐sellers. Finally, the physicians' workshop drew on findings from all prior sessions, allowing participants to situate their perspectives within the broader stakeholder context.

Each workshop lasted approximately two hours and was facilitated by a primary facilitator supported by two co‐facilitators, who assisted in guiding activities, ensuring smooth implementation, and documenting discussions. Written informed consent was obtained in the local language before participation. Participants were assured that involvement would not pose professional, financial, reputational, or legal risks. Audio recordings, photographs, and videos were taken only with prior permission, and all identifiable information was removed and made accessible only to the research team.

**Photo 3:**
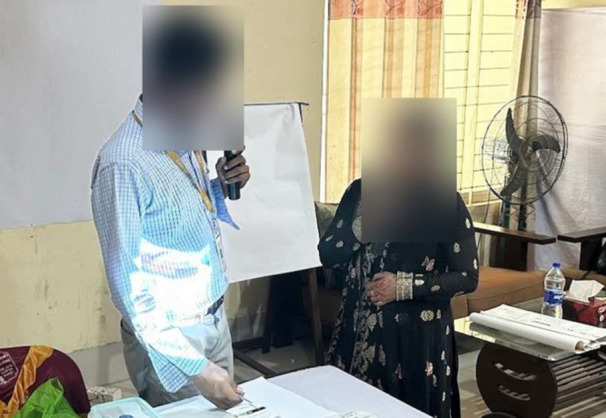
Role‐play exercises simulating typical rural health‐seeking interactions in community pharmacies.

**Photo 4:**
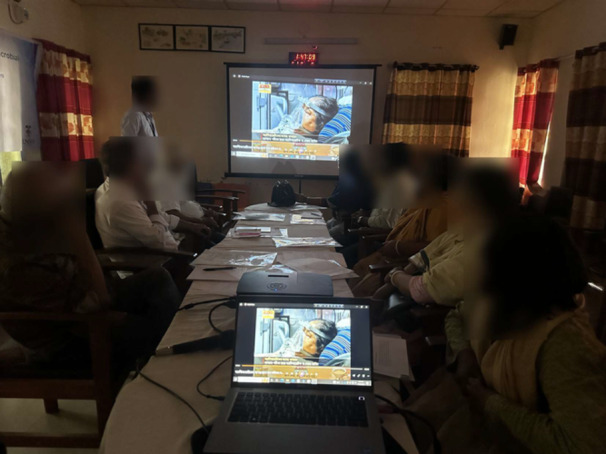
Showing a brief video to registered physicians on the current antibiotic use scenario in Bangladesh.

**Photo 5:**
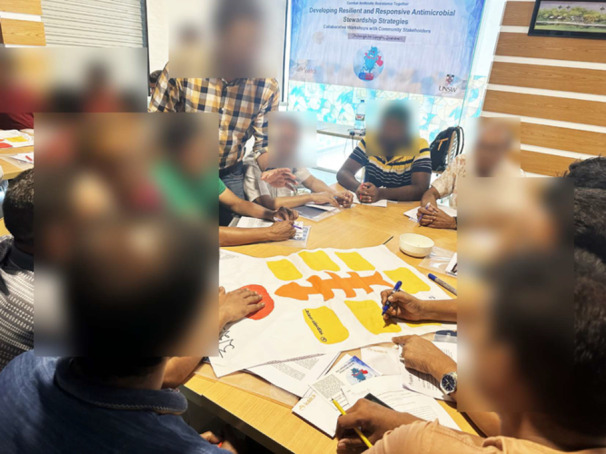
Pharmacy drug‐sellers group discussing factors contributing to inappropriate antibiotic use during fishbone analysis.

**Photo 6:**
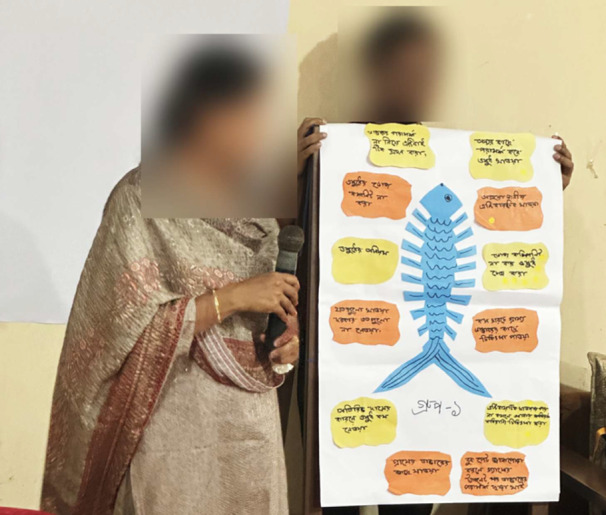
A woman from the antibiotic customer group presents the outcomes of the group discussion.

The sessions were structured into three main phases:

In Activity 1, participants identified factors contributing to inappropriate antibiotic use. Sessions began with a role play simulating a typical rural healthcare‐seeking scenario in Bangladesh (Photo 3), where participants demonstrated symptom reporting, pharmacy visits, and requests for quick relief medicines. Registered physicians did not participate in the role play because of time constraints and instead viewed a brief video illustrating the current antibiotic use scenario in Bangladesh as an ice‐breaking activity (Photo 4). Participants were then divided into subgroups and used a fishbone diagram to identify key behaviours and contextual factors contributing to antibiotic misuse (Photos 5 and 6).

**Photo 7:**
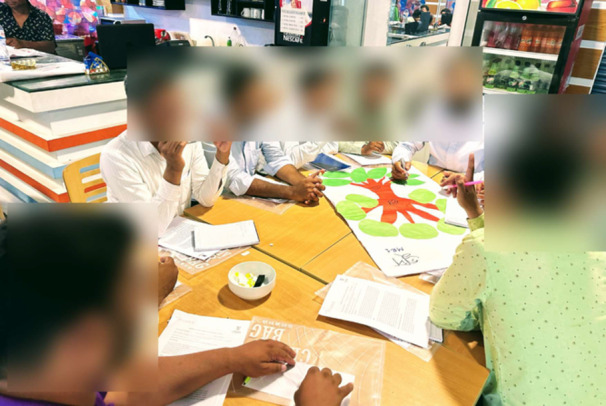
The pharmaceutical sales representative group discussed strategies for identified problems and completed the activity posters.

**Photo 8:**
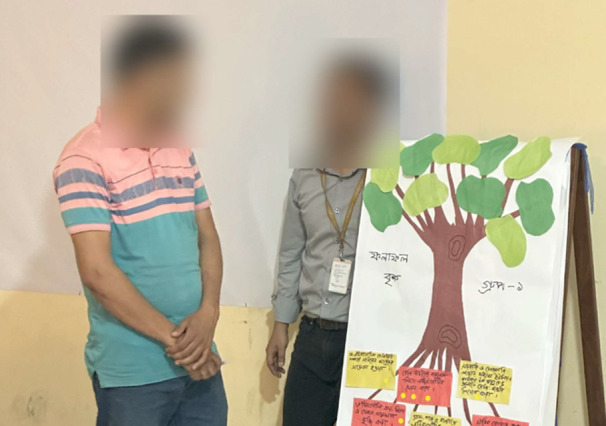
A drug‐seller member presented the drug‐sellers group's proposed strategies.

In Activity 2, participants worked in subgroups to propose practical and role‐specific strategies to address the identified problems. Proposed strategies were documented using an impact tree matrix and categorized across systemic, organizational, community, and individual levels (Photos 7 and 8).

**Photo 9:**
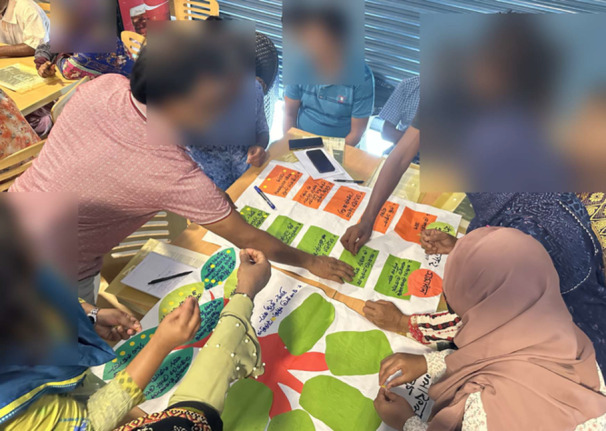
Participants were identifying implementation challenges.

**Photo 10:**
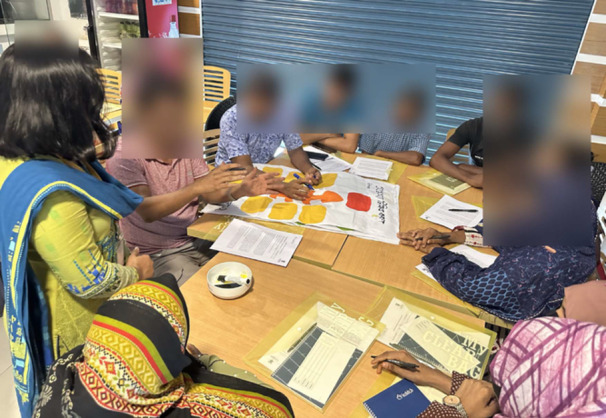
A co‐facilitator was guiding the antibiotic user's group on how to conduct the group exercise.

In Activity 3, participants identified anticipated implementation challenges related to the proposed strategies and documented them on poster paper for further discussion. Participants also reflected on the potential impact of the proposed strategies and barriers to implementation, which were informally documented by co‐facilitators. After each activity, one participant from each subgroup presented the discussion outcomes, and feedback was collected from all participants (Photos 9 and 10).

Within each workshop, participants expressed both agreement and differing perspectives. When disagreements arose, facilitators encouraged discussion until consensus was reached. The agreed decisions were then documented on the respective fishbone diagrams, impact trees, and implementation challenges matrices. Consequently, all workshop outputs reflected the participants' consensus views.

### 2.4 Data Analysis

All workshop materials were collected and reviewed, including session notes, audio recordings, photographs, and outputs from group activities, such as fishbone analyses, impact tree analyses, and implementation challenge matrices. At the conclusion of each workshop, participant groups presented their findings, including identified problems, proposed solutions, and anticipated implementation challenges. These presentations were audio‐recorded and subsequently transcribed. During analysis, workshop outputs were cross‐checked against the transcripts to ensure consistency and completeness of interpretation. Participants' demographic characteristics, including gender, age, educational attainment, type of involvement, years of experience, and institutional affiliation (where applicable), were extracted from registration and attendance records and are presented in Table S1.

**Figure 1:**
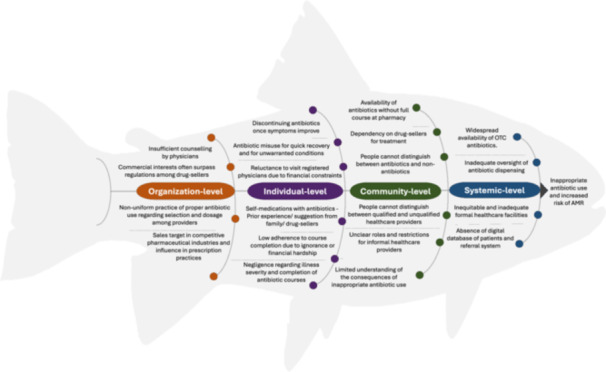
Problem identification in the Ishikawa diagram.

**Figure 2:**
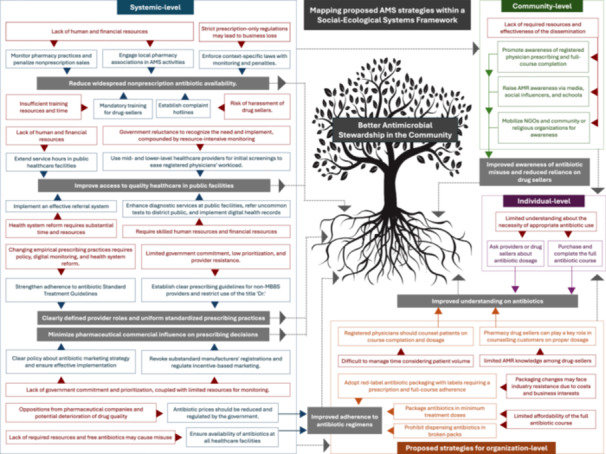
Proposed AMS strategies and anticipated implementation challenges. AMS, antimicrobial stewardship.

Analysis was conducted by the research team and began by compiling and synthesizing findings from similar stakeholder groups across the two workshops. The synthesized findings were then integrated into a consolidated table that captured identified drivers of nonprescription and inappropriate antibiotic use, proposed AMS strategies, and anticipated implementation challenges. For each proposed strategy, the implementation level identified by participants was recorded in the “Strategy implementation level” column, and the stakeholder group or groups proposing each strategy were documented in the “Strategy proposed by” column. Blank cells indicate that a strategy was not proposed by that stakeholder group. The consolidated findings were subsequently summarized in Figure 1, 1, Figure 2, 2, and Table S2. Participant reflections documented by co‐facilitators were incorporated, where relevant, to support the interpretation of the findings. The findings were then organized according to the four levels of the Social Ecological Systems Framework, namely, the systemic, organizational, community, and individual levels [36]. Given that the primary objective of the codesign process was to develop context‐specific AMS strategies, the analysis placed greater emphasis on proposed strategies and anticipated implementation challenges than on problem identification alone.

To contextualize the proposed strategies within existing national priorities, all AMS strategies identified in the consolidated table were compared with the Bangladesh National Action Plan (NAP) for Antimicrobial Resistance Containment 2023–2028 [37]. The results are presented in Table S3, which indicates whether each strategy was directly included, indirectly supported, or not addressed in the NAP, together with the relevant section and page references. Implementation priority was assigned based on each strategy's prominence in the NAP.

## 3 Results

Participant groups varied in age, gender, educational background, and professional roles, with detailed demographic characteristics presented in Table S1. The antibiotic user group comprised individuals of diverse ages, educational backgrounds, and socioeconomic backgrounds. The registered physician group included both male and female participants recruited from public and private healthcare facilities, with varying years of clinical experience. All participants in the drug‐seller and pharmaceutical sales representative groups were male. Drug‐sellers included both pharmacy owners and sales staff working in registered and unregistered pharmacies and represented a range of ages, educational backgrounds, and training experiences. Pharmaceutical sales representatives were relatively similar in educational background but varied in their years of professional experience.

### 3.1 Proposed Systemic‐Level Strategies

#### 3.1.1 Regulating Over‐the‐Counter (OTC) Antibiotic Availability

Participants across all stakeholder groups identified the widespread availability of OTC antibiotics as a key driver of misuse. They attributed this to weak enforcement of prescription regulations and the commercial interests of drug‐sellers. All stakeholder groups recommended developing context‐specific regulations, supported by strict monitoring, penalties for noncompliance, and a hotline for reporting violations. Most participants also recommended mandatory training for all drug‐sellers on antibiotic dispensing and patient counselling, particularly regarding dosage instructions. In addition, many participants suggested involving district‐level pharmacy owners' associations in AMS awareness‐raising and engagement activities.

Participants also identified several anticipated challenges to implementation. These included limited government human and financial resources for monitoring, training, and engaging district‐level pharmacy owners' associations. Participants noted that restricting antibiotic sales to prescription only could result in financial losses for pharmacy owners, which they perceived as a potential barrier to compliance. They also expressed concerns that both dispensers and customers might seek ways to circumvent the regulations. Although participants considered a complaint hotline a potential mechanism for reporting noncompliance, some raised concerns that it could be misused and lead to harassment of drug‐sellers. During the workshops, drug‐sellers further noted that if regulations were enforced uniformly across all pharmacies, opportunities to bypass the rules would be reduced, thereby minimizing the risk of losing customers and reducing concerns about business sustainability.

#### 3.1.2 Improve Access to Quality Healthcare

Participants from antibiotic users, sales representatives, and drug‐sellers noted that limited access to public healthcare—due to long wait times, poor quality, and lack of diagnostic facilities—discourages use of these services. High out‐of‐pocket expenses in private healthcare further push individuals toward symptomatic treatment from drug‐sellers. Inadequate counselling by formal providers also contributes to poor understanding of proper antibiotic use and dosage. Registered physicians noted that many routine visits could be managed by mid‐ and lower‐level staff, such as Sub‐Assistant Community Medical Officer (SACMOs) and paramedics, reducing physician workload; however, their underutilization results in long waits and limited counselling time.

To address these issues, most participant groups, except registered physicians, proposed extending service hours in public facilities to improve access for lower‐income individuals occupied during the day. Physicians emphasized the need for an effective referral system and better use of mid‐ and lower‐level staff for initial screening to reduce patient load and waiting times, as well as the implementation of digital patient records with identifiers (e.g., national ID) to guide appropriate antibiotic selection. All groups suggested making common diagnostic tests available in public facilities, with less common tests referred to district‐level public labs. Registered physicians mentioned that public‐private partnerships, such as health insurance or co‐contributory schemes used in other countries, could enable affordable referrals for tests unavailable in public healthcare facilities.

However, these strategies face significant challenges, including the need for substantial skilled human and financial resources. Registered physicians also pointed to limited government willingness, human resource shortages, and systemic constraints as major barriers to implementing referral systems, digital databases, active diagnostic support, extended service hours, and improved counselling. They noted that health system reforms in resource‐constrained settings are often challenging, time‐consuming, and resource‐intensive, with limited political commitment.

#### 3.1.3 Ensuring Access to Antibiotics to Support Course Completion

Participants across groups noted that antibiotics are often expensive for low‐income individuals, leading many to discontinue treatment once symptoms improve due to cost and a lack of understanding about the consequences of incomplete use. Antibiotic users and drug‐sellers suggested government regulation to reduce prices and provide common antibiotics free at public facilities to improve adherence. However, drug‐sellers and pharmaceutical sales representatives cautioned that major pharmaceutical companies might oppose price cuts, and lesser‐known companies could produce low‐quality antibiotics to maintain profits. Registered physicians acknowledged that many common antibiotics are available in public facilities but inconsistently and warned that increasing availability alone might encourage misuse; instead, they emphasized the need to educate people on appropriate use.

#### 3.1.4 Clarifying Provider Roles and Standardizing Prescribing

Participants reported that people often cannot distinguish qualified from unqualified providers. Registered physician groups and pharmaceutical sales representatives reported that many village doctors and lower‐level health staff (e.g., SACMOs and paramedics) practice empirical treatment and are perceived as doctors for using the “Dr.” title and printing prescriptions.

Registered physicians emphasized the need for clear policies defining the roles and authorized treatments of qualified versus nonqualified providers, along with strong enforcement to prevent misuse of the “Dr.” title. They noted that despite existing policies, enforcement remains weak, and recent government efforts have faced protests from mid‐ and lower‐level staff.

Physicians also highlighted that patients' reluctance to undergo diagnostics and demand for medications often drives empirical antibiotic prescribing and inconsistent practices. Many physicians are unaware of or do not follow standard treatment protocols. Physicians suggested that governments and healthcare institutions ensure physicians are informed of and compliant with guidelines. They noted that addressing these entrenched empirical practices and ensuring compliance would require clear policies, intensive monitoring, and comprehensive health system reforms.

#### 3.1.5 Regulatory Oversight on Antibiotic Marketing

Participants identified inadequate oversight of antibiotic dispensing and aggressive, profit‐driven pharmaceutical marketing as significant contributors to antibiotic misuse. Registered physicians, drug‐sellers, and sales representatives acknowledged that sales targets drive aggressive marketing. To address this, participants recommended government monitoring and clear guidelines on antibiotic marketing practices, including defining permissible activities and ensuring compliance. Participants highlighted that many companies operate without proper standards or registration, leading to market overcrowding and increased competition. Small pharmaceutical companies often market substandard antibiotics, raising resistance risks, and participants stressed the need for government action to cancel the operations of companies that fail to meet standards. Drug‐sellers reported that pharmaceutical companies frequently influence registered physicians by offering incentives to promote their products. They proposed promoting generic antibiotics to reduce this influence. However, registered physicians and sales representatives opposed introducing generic names, arguing that physicians are well trained to avoid unnecessary antibiotic use, and that promotional activities mainly affect brand choice rather than misuse. They warned that if generics are introduced, drug‐sellers might favour lesser‐known, lower‐quality brands for higher profits, further risking substandard antibiotic circulation. All groups emphasized the need for government action to eliminate noncompliant companies. Despite these recommendations, implementation faces challenges due to limited government commitment, prioritization, and insufficient resources for effective monitoring.

### 3.2 Proposed Organization‐Level Strategies

#### 3.2.1 Right Drug, Right Dose, and Right Duration

Across all participant groups, a common concern was that many people, including drug‐sellers, cannot distinguish antibiotics from nonantibiotics and often use them interchangeably for unwarranted conditions without understanding the consequences or the necessity of taking the antibiotic for the correct duration. Physicians, drug‐sellers, and pharmaceutical sales representatives reported that some widely used antibiotics are not recognized as such, even by drug‐sellers. In addition, registered physicians, drug‐sellers, and pharmaceutical sales representatives also reported that an unregulated market characterized by numerous pharmaceutical companies and overcrowded pharmacies fosters intense competition. This environment leads to unethical marketing practices that emphasize antibiotic effectiveness while neglecting the consequences of misuse or inappropriate use. Sales representatives admitted that pressure to meet sales targets drives aggressive marketing tactics.

To address confusion around antibiotic identification, physicians, drug‐sellers, and pharmaceutical sales representatives proposed introducing clear antibiotic packaging that includes a specific colour code. This measure aims to help both the public and drug‐sellers differentiate antibiotics from other medicines and reduce misuse. Additionally, drug‐sellers, registered physicians, and pharmaceutical sales representatives suggested that the government mandate packaging containing the full minimum antibiotic course, preventing pharmacies from dispensing partial courses. To further mitigate misuse and unethical marketing, these groups recommended clear labelling with colour codes and warnings indicating that antibiotics require a registered physician's prescription. Pharmaceutical sales representatives noted that some companies have begun introducing red‐label packaging with precautionary messages. Packaging changes involve significant time and financial investment by pharmaceutical companies. Widespread adoption of improved packaging, such as red‐label antibiotics, has been slow, especially among smaller companies, due to existing stock and repackaging costs. Even with mandated full‐course dispensing, participants recognized that low‐income individuals may still struggle to afford the recommended dose due to daily income constraints. However, drug‐sellers indicated that selling the recommended dose offset losses from restricting sales to prescriptions only since per‐unit profits would increase. Participants noted that ensuring effective training for pharmacy drug‐sellers would depend on government enforcement and cooperation from pharmacy owners. They further noted that addressing unethical marketing practices and the sale of substandard antibiotics would require firm regulatory oversight and government intervention in the pharmaceutical market.

#### 3.2.2 Enhance Patient Counselling and Engage Pharmacy Drug‐Sellers in Dosage Guidance

Participants reported that people commonly expect antibiotics to act quickly and often stop taking them once symptoms subside, primarily due to a lack of knowledge about the risks of incomplete treatment. Despite frequent visits to pharmacies for nonsevere illnesses, people tend to value advice from registered physicians more highly. However, increased patient counselling by physicians is currently limited due to high doctor‐to‐patient ratios. To support the quality use of medications, participants recommended increased patient counselling by physicians. Additionally, pharmacy drug‐sellers were identified as potential counsellors for dosage guidance, provided they receive proper training on antibiotic dispensing and AMR. Participants suggested that the government mandate such training and ensure support from pharmacy owners to implement it effectively. Participants noted that enhancing the counselling role of pharmacy drug‐sellers would depend on the availability and quality of their training in antibiotic stewardship and resistance. They noted that effective and mandatory training would require government enforcement and cooperation from pharmacy owners.

### 3.3 Proposed Community‐Level Strategies

Lack of community awareness about proper antibiotic use and its consequences, misuse for quick recovery and unwarranted conditions, and reliance on drug‐sellers are key drivers of antibiotic misuse. Participants felt that the term antibiotic resistance was poorly understood by both the community and many health professionals. Misuse is fuelled by misinformation, overreliance on drug‐sellers, and confusion about what an antibiotic is. To address this, they recommended comprehensive mass awareness campaigns targeting diverse socioeconomic groups to highlight the risks of antibiotic misuse. These campaigns should leverage social media, television, mobile messaging, school curricula, and healthcare settings, emphasizing that antibiotics must only be prescribed by registered physicians. Participants noted that successful implementation would require coordinated efforts from governments, organizations, communities, and individuals. Additionally, nongovernment organizations (NGOs), community‐based groups, and social or religious institutions were identified as key partners to support these initiatives alongside government programmes. Participants acknowledged challenges such as limited resources for awareness efforts and scepticism about the effectiveness of messaging, noting that entrenched health‐seeking behaviours are difficult to change. They suggested delivering messages through entertaining formats or via public figures, social influencers, and celebrities to capture attention, as engagement tends to wane quickly without compelling content. There was broad consensus on integrating antibiotic risk education into secondary school curricula, particularly in health or physical education, to promote long‐term understanding.

Although community‐level interventions are important, all groups agreed that awareness alone is insufficient without strong regulatory and monitoring frameworks supported at the systemic level. For example, some drug‐sellers referenced the COVID‐19 lockdown, where initial resistance to mask‐wearing and handwashing shifted to widespread compliance due to fear and strict government enforcement, but these behaviours faded once oversight lessened. Therefore, participants stressed that sustained public education on the dangers of arbitrary antibiotic use must be supported by effective legal and regulatory measures to ensure lasting behaviour change.

### 3.4 Proposed Individual‐Level Strategies

Participants across groups acknowledged that final responsibility for appropriate antibiotic use lies with individuals and their families, but emphasized that without community‐level adoption, individuals are unlikely to change their practices. They highlighted that self‐medication, often based on previous recovery or advice from friends and family, and the inability to distinguish antibiotics from nonantibiotics, leading to interchangeable use, significantly drive antibiotic misuse. Participants suggested that, at the individual and interpersonal levels, people should ask whether medications contain antibiotics, confirm the duration when consulting healthcare providers or purchasing antibiotics, and complete the full course. However, lack of motivation, ignorance, and inability to afford the full course remain major barriers. Participants agreed that greater mass awareness and observing these behaviours within the community would promote wider acceptance and adherence.

## 4 Discussion

In this codesign research, participants from all groups proposed AMS strategies across systemic, organizational, community, and individual levels to improve health‐seeking behaviours and antibiotic use through active stakeholder engagement. Most strategies focused on systemic reform, emphasizing policy development, law enforcement, and health system strengthening. Key systemic issues identified included widespread availability of OTC antibiotics, limited access to formal healthcare, inadequate oversight of antibiotic marketing and dispensing, and unclear prescribing roles among healthcare providers. To address these, stakeholders recommended developing context‐specific policies, regulating pharmacy practices, strengthening diagnostics, implementing digital referral systems, optimizing the use of the healthcare workforce, clarifying provider qualifications, and enforcing Standard Treatment Guidelines (STGs). These recommendations align with global evidence that AMR in LMICs stems from structural challenges, such as poverty, high out‐of‐pocket costs, weak infrastructure, limited diagnostics, and poor regulation—factors that drive self‐medication and inappropriate antibiotic use [38, 39]. A scoping review further emphasizes that irrational antibiotic use within Bangladesh's unregulated pluralistic health system intensifies AMR, reinforcing the urgent need for comprehensive policy and practice reforms [40]. The NAP for AMR Containment in Bangladesh 2023–2028 also highlights these issues as key activities with high implementation priority [37]. Strengthening health systems, particularly pharmaceutical components, is essential for effective AMS, ensuring access to safe, affordable medicines and enabling implementation at national and facility levels [41, 42, 43].

However, systemic reforms are often hindered by limited financial and human resources, political inertia, and conflicts of interest with pharmaceutical companies—reflecting broader structural barriers in LMICs [44]. A joint report by WHO and Global Antibiotic Research and Development Partnership notes that National Regulatory Authorities (NRAs) in LMICs frequently lack the capacity to manage antibiotic shortages and misuse, making the strengthening of core NRA functions a priority, followed by policy enforcement and systemic reforms such as procurement regulation and advanced market surveillance through track‐and‐trace systems [45]. Broader health system reforms also address critical factors like infrastructure, policy, socioeconomic conditions, clinical practices, regulation, and global trade, as emphasized in recent AMR overviews [46]. Similar strategies in Central Asia advocate monitoring antibiotic consumption, strengthening diagnostics, implementing digital referral systems, and enforcing STGs [47]. In LMICs, pharmaceutical companies' sales‐driven priorities often conflict with AMS objectives, with declining antibiotic profitability and aggressive marketing contributing to reluctance in adopting restrictive policies [48, 49]. Market pressures on pharmacies and drug‐sellers further promote overdispensing, undermining stewardship, while policy analyses identify commercial resistance and weak enforcement as key barriers to AMS implementation [50, 51]. Weak governance and inadequate legal frameworks limit pharmacists' ability to enforce proper dispensing [52], while many community pharmacies operate without qualified staff and sell substandard or falsified medicines. Resource constraints and fragile supply chains further hinder enforcement [5, 53, 54]. Southeast Asian countries recommend engaging drug‐sellers in AMR action plans, strengthening regulation and enforcement, enhancing provider training, promoting pharmacist‐led stewardship initiatives, and conducting public awareness campaigns to reduce antibiotic misuse [55, 56, 57, 58, 59].

Participants across groups reported widespread confusion between antibiotics and nonantibiotics, leading to misuse and poor adherence. They recommended organizational strategies such as clear, colour‐coded antibiotic packaging with labels. Specifically, red‐label packaging to deter nonprescription sales, minimum dosage packs to ensure appropriate dosage, enhanced patient counselling, and stronger government oversight of antibiotic marketing and sales were proposed—measures well supported in AMS literature [39, 51]. In Bangladesh, a 2021 DGDA baseline survey found many pharmacy retailers unable to recognize antibiotics, highlighting the need for packaging reforms to improve drug identification [14]. Supporting this, the AntiBiotic ACcess and USe II study (including Bangladesh sites) showed that improving the physical appearance of antibiotics enhances appropriate use by community members and healthcare providers, focusing on better prescription‐only medicine identification, reducing self‐medication, and raising awareness [60, 61]. Similarly, India's 2016 “Red Line” campaign introduced a vertical red line on prescription‐only packaging to curb self‐medication and improve awareness [62]. Though recognized as promising in the global review on AMR [63], evaluations revealed low awareness among healthcare professionals and patients, indicating that visual cues alone require broader educational efforts to be effective [64]. However, challenges persist, especially in South Asia, where red‐label packaging and similar stewardship measures face resistance from pharmaceutical companies concerned about increased costs, reduced sales, and profitability [14, 65, 66]. Commercial incentives and marketing practices in LMICs often conflict with stewardship goals, while industry lobbying and weak enforcement hinder regulatory uptake [48, 67].

In Bangladesh, the DGDA, supported by WHO, mandated red‐label antibiotic packaging with clear warnings as a national AMS intervention from December 2022. However, major pharmaceutical companies raised concerns about operational costs and market impact, reflecting ongoing tensions between stewardship objectives and commercial interests [14]. Red‐label packaging aims to deter nonprescription sales by signalling high‐risk medications, while minimum dosage packs help ensure treatment completion [20, 34]. While minimum dosage packaging has been effective in many LMICs [68], financial hardship can prevent low‐income patients from purchasing a full course at once, contributing to inequitable access and potential treatment failure [68, 69, 70]. This highlights the need for innovative models to ensure affordability and access to antibiotics in resource‐limited settings [68, 71]. Enhanced patient counselling and education are vital, with pharmacists and drug‐sellers playing key roles in improving adherence and awareness [5, 50]. Gaps in provider training and the prevalence of informal providers, particularly in rural areas, also limit effective patient counselling [68]. A recent study on community antibiotic use recommends strengthening provider‐patient communication, which positively influences AMS outcomes [32].

All participant groups identified a lack of awareness about the consequences of antibiotic misuse as a key barrier to effective AMS among patients and providers. Misconceptions, existing health‐seeking behaviours, high self‐medication rates, and reliance on community‐pharmacy drug‐sellers drive inappropriate use, including unnecessary treatments. At the community level, participants recommended mass awareness campaigns across diverse communication channels, the integration of educational messages into textbooks and healthcare settings, and the involvement of community organizations and NGOs to disseminate these messages. At the individual level, patients were encouraged to actively ask healthcare providers and pharmacists about antibiotic content, dosage, and duration, and to complete prescribed courses—though perceived these behaviours rely on increased community awareness.

These findings align with WHO recommendations for tailored public engagement and media outreach to reduce nonprescription antibiotic use [72]. Supporting this, a systematic review confirms that mass media campaigns via television, radio, and social media effectively improve public knowledge, attitudes, and behaviours concerning antibiotic use [73]. In Southeast Asia, the Community Engagement for AMR network developed culturally tailored interventions, including educational campaigns, community workshops, and local policy initiatives, through active community engagement [74]. Addressing broader social determinants through community engagement and context‐specific policies further fosters responsible antibiotic use and sustained behaviour change [75]. In Bangladesh, WHO‐supported initiatives combine public awareness with data‐driven regulatory measures to strengthen stewardship [14]. Recent studies also advocate targeted campaigns to raise awareness about the risks of self‐medication and promote appropriate antibiotic use [76]. However, codesign research highlights key implementation challenges for mass awareness‐driven behaviour change, notably entrenched perceptions and health‐seeking practices for nonsevere conditions, alongside limited resources for widespread dissemination. These challenges mirror existing literature documenting a widespread lack of understanding of AMR and appropriate antibiotic use in Bangladesh [77], compounded by socioeconomic barriers, such as poverty and limited access to healthcare, which drive self‐medication, especially in rural areas [76].

### 4.1 Limitations

This study has several limitations to consider when interpreting the findings. Most participant groups, except registered physicians, showed limited knowledge of AMR and were largely unaware of existing AMR policies or regulatory action in Bangladesh. Rather than a study weakness, this reflects the actual knowledge landscape among stakeholders engaged in antibiotic use and dispensing, and underscores the real‐world context the intervention aims to address. Similarly, the considerable heterogeneity within groups, particularly among antibiotic users and drug‐sellers who varied in education, age, socioeconomic status, and dispensing experience, mirrors the diversity of actors within Bangladesh's pluralistic health system rather than a sampling weakness.

This variation in prior knowledge nonetheless likely shaped the sophistication of the strategies proposed, with more knowledgeable participants tending to introduce more developed ideas. Facilitators actively managed group discussions, encouraging quieter participants to contribute and guiding disagreements toward consensus before outputs were recorded on the fishbone diagrams, impact trees, and implementation challenges matrices. Even with this facilitation, the influence of more vocal or senior participants on the final consensus cannot be entirely ruled out, particularly given the power differentials among stakeholders, such as physicians, pharmaceutical representatives, drug‐sellers, and antibiotic users. The decision to conduct some workshops separately by stakeholder type and others as mixed groups was intended to balance candid expression against cross‐group learning, but this design choice may still have influenced the depth and framing of recommendations in each format. Because the analysis drew on outputs from all groups, recommendations on certain topics were disproportionately shaped by groups with greater exposure to those issues.

The study was conducted in selected rural settings, and findings may not be directly transferable to urban contexts or regions with different health system configurations, provider compositions, or regulatory environments. Finally, although participants identified contextually relevant strategies and anticipated implementation challenges, feasibility was discussed rather than empirically tested; establishing actual feasibility would require piloting and further engagement with policymakers, regulatory authorities, and health system decision‐makers.

## 5 Conclusion

AMR in LMICs is a complex, multisectoral challenge requiring coordinated policy and practice reforms. Bangladesh's AMR NAP continues to face weak intersectoral coordination, inadequate regulatory enforcement, and limited resources that impede effective AMS [78]. Building on these documented challenges, this study presents a codesigned and prioritized set of strategies spanning the system, organizational, community, and individual levels, along with the implementation barriers perceived by registered physicians, pharmaceutical sales representatives, drug sellers, and antibiotic users involved in antibiotic use and dispensing at the community level. This multiactor perspective extends beyond earlier calls for multilevel AMS by clarifying which strategies stakeholders themselves consider workable, the enabling conditions that support their implementation, and where resistance, cost, and enforcement gaps are likely to arise in practice. Consistent with previous findings, stakeholders emphasized that reforms must be consistently enforced and aligned with local economic realities, including a clearer understanding of provider behaviours, economic priorities, and consumer needs, particularly within drug shops [6]. Efforts to curb irrational antibiotic use and OTC sales are unlikely to succeed without health system strengthening and government‐led policy enforcement, since sustained behaviour change depends on an enabling environment supported by robust AMR governance [51]. Aligning these codesigned strategies with local economic realities through coordinated, context‐sensitive approaches remains essential to curb AMR and ensure equitable access to safe antibiotics in resource‐limited settings, such as Bangladesh.

## Author Contributions

**Abdullah Al Masud:** conceptualization, investigation, writing – original draft, methodology, validation, visualization, writing – review and editing, software, formal analysis, project administration, data curation, resources. **Kamal Ibne Amin Chowdhury:** investigation, funding acquisition, methodology, supervision, resources, project administration. **Ramesh Lahiru Walpola:** conceptualization, funding acquisition, methodology, supervision, writing – review and editing. **S. M. Zafar Shafique:** investigation, formal analysis, supervision, data curation, resources, project administration, methodology, validation. **Tamanna Sultana:** investigation, formal analysis, project administration, data curation, validation. **Maria Akter:** investigation, validation, formal analysis, project administration, data curation. **Nisharggo Niloy:** investigation, project administration, resources, formal analysis. **Md. Saiful Islam:** conceptualization, funding acquisition, supervision, resources, writing – review and editing, methodology. **Holly Seale:** conceptualization, funding acquisition, writing – review and editing, visualization, validation, methodology, supervision, resources, formal analysis, project administration.

## Ethics Statement

Ethics approval for the study was obtained from the University of New South Wales (UNSW) Human Research Ethics Committee (iRECS4996) and the Institutional Review Board of ICDDR,B (PR‐24024). Written informed consent was obtained from all participants on the day of the workshop. Participants were compensated according to ICDDR,B's incentive structure, which was reviewed and approved by the Institutional Review Board (IRB) before the study commenced.

## Conflicts of Interest

The authors declare no conflicts of interest. Any potential competing interests have been acknowledged and managed to maintain the study's integrity and impartiality.

## Declaration on the Use of AI‐Assisted Tools

During the preparation of this manuscript, AI‐assisted tools were used only for language editing and fixing grammatical errors.

## Supporting information


Supporting File

## Data Availability

The data that support the findings of this study are available on request from the corresponding author, in accordance with the data‐sharing policies of the University of New South Wales and ICDDR,B. Supporting Information files, including supporting data and summaries, are available. The data are not publicly available due to privacy or ethical restrictions.
