# Supplementary material for: A Codesigned Approach to Develop Tailored Antimicrobial Stewardship Strategies in Bangladesh: A Social–Ecological Systems Framework Analysis
Source: Health Expect. 2026 Aug 17;29(4):e70822. doi: 10.1111/hex.70822 (PMC13482172; doi:10.1111/hex.70822)
Supplement: Supplementary file 1 [file HEX-29-e70822-s001.pdf]

Supplementary materials

Table 1: Demographic characteristics of workshop participants across multiple groups

Characteristics	Registered Physician		Sales-representatives		Pharmacy drug-sellers		Antibiotic-users	
	Rural 1	Rural 2	Rural 1	Rural 2	Rural 1	Rural 2	Rural 1	Rural 2
	n/8 (%)	n/4 (%)	n/30 (%)	n/17 (%)	n/22 (%)	n/15 (%)	n/18 (%)	n/21 (%)
**Sex**								
Male	4 (50%)	3 (75%)	30 (100%)	17 (100%)	22 (100%)	15 (100%)	11 (61%)	13 (62%)
Female	4 (50%)	1 (25%)	0 (0%)	0 (0%)	0 (0%)	0 (0%)	7 (39%)	8 (38%)
**Age**								
18-30 years	1 (13%)	3 (75%)	13 (43%)	3 (18%)	8 (36%)	5 (33%)	N/A	9 (43%)
31-40 years	1 (13%)	1 (25%)	13 (43%)	13 (76%)	3 (14%)	4 (27%)	N/A	6 (29%)
41-50 years	1 (13%)	0 (0%)	3 (10%)	1 (6%)	6 (27%)	4 (27%)	N/A	4 (19%)
51 and above	5 (63%)	0 (0%)	1 (3%)	0 (0%)	5 (23%)	2 (13%)	N/A	2 (10%)
**Education**								
No formal education	N/A	N/A	0 (0%)	0 (0%)	0 (0%)	0 (0%)	0 (0%)	0 (0%)
≤ Grade 5 (Primary)	N/A	N/A	0 (0%)	0 (0%)	0 (0%)	0 (0%)	3 (17%)	5 (24%)
≤ Grade 10 (SSC)	N/A	N/A	0 (0%)	0 (0%)	3 (14%)	1 (7%)	6 (33%)	6 (29%)
Completed grade 12 (HSC)	N/A	N/A	1 (3%)	0 (0%)	9 (41%)	5 (33%)	2 (11%)	4 (19%)
Graduation and above	8 (100%)	4 (100%)	29 (97%)	17 (100%)	10 (45%)	9 (60%)	7 (39%)	6 (29%)
MBBS	6 (75%)	4 (100%)	N/A	N/A	N/A	N/A	N/A	N/A
MBBS, FCPS	2 (25%)	0 (0%)	N/A	N/A	N/A	N/A	N/A	N/A
**Type of involvement**								
General Physician/Medical officer	5 (63%)	4 (100%)	N/A	N/A	N/A	N/A	N/A	N/A
Surgeon	3 (38%)	0 (0%)	N/A	N/A	N/A	N/A	N/A	N/A
Medical promotion officer	N/A	N/A	24 (80%)	14 (82%)	N/A	N/A	N/A	N/A

Senior Medical Promotion Officer	N/A	N/A	6 (20%)	3 (18%)	N/A	N/A	N/A	N/A
Pharmacy owner	N/A	N/A	N/A	N/A	10 (45%)	0 (0%)	N/A	N/A
Drug-sellers	N/A	N/A	N/A	N/A	10 (44%)	7 (47%)	N/A	N/A
Village doctor cum pharmacy owner	N/A	N/A	N/A	N/A	2 (9%)	8 (53%)	N/A	N/A
**Facility type**								
Public healthcare facility	4 (50%)	2 (50%)	N/A	N/A	N/A	N/A	N/A	N/A
Private healthcare facility	4 (50%)	2 (50%)	N/A	N/A	N/A	N/A	N/A	N/A
Pharmaceutical Company	N/A	N/A	30 (100%)	17 (100%)	N/A	N/A	N/A	N/A
Community pharmacy	N/A	N/A	N/A	N/A	22 (100%)	15 (100%)	N/A	N/A
**Work experience in the relevant field**								
≤ 2 years	0 (0%)	0 (0%)	8 (27%)	3 (18%)	0 (0%)	3 (20%)	N/A	N/A
3 to 5 years	1 (13%)	3 (75%)	11 (37%)	6 (35%)	6 (27%)	4 (27%)	N/A	N/A
6 to 10 years	0 (0%)	1 (25%)	6 (20%)	5 (29%)	2 (9%)	0 (0%)	N/A	N/A
11 to 20 years	2 (25%)	0 (0%)	3 (10%)	3 (18%)	4 (18%)	7 (47%)	N/A	N/A
21 years above	5 (63%)	0 (0%)	2 (7%)	**0 (0%)**	10 (45%)	1 (7%)	N/A	N/A

Table 2: Consolidated table of co-design workshop outcomes across all participant groups

**Identifies key problems** (Activity 1)	**Proposed AMS strategies** (Activity 2)	**Anticipated implementation challenges** (Activity 3)	**Strategy implementation level**				**Strategy proposed by**			
			**Systemic level**	**Organization level**	**Community level**	**Individual level**	**Drug-sellers**	**Sales rep.**	**Reg. physicians**	**Antibiotic users**
**Over-the-counter antibiotic availability:**<br>▪ Widespread OTC antibiotics widely available.<br>▪ Inadequate oversight of antibiotic dispensing.<br>▪ Commercial interests override rules.<br>▪ Limited understanding on antibiotics among pharmacy drug-sellers	Formulate context-specific laws with regular monitoring and justified penalties	Lack of required human and financial resources from government.	✓				✓	✓	✓	✓
	Monitor pharmacy dispensing practices regularly and enforce appropriate penalties for non-prescription dispensing	Strict prescription-only antibiotic sales may lead to business losses for pharmacy owners.<br>Both dispenser and customers may attempt to violate the regulations	✓				✓	✓	✓	✓
	Implement a mandatory free short course on antibiotic dispensing for drug-sellers and ensure all complete the training.	Resources needed for training and pharmacy owners' flexibility to allocate time for staff training.	✓				✓	✓	✓	
	Establish a dedicated hotline for complaints.	Many drug-sellers may become victims of harassment	✓				✓			✓
	Engage district-level pharmacy associations to develop strategies and clarify their role in AMS.	Lack of required human and financial resources from government.	✓				✓	✓	✓	

**Limited awareness of antibiotic misuse consequences and reliance on drug-sellers:**<br>▪ Antibiotic misuse for quick recovery and for unwarranted conditions<br>▪ Dependency on drug-sellers for treatment<br>▪ Lack of mass awareness about appropriate antibiotics and consequences of misuse.	Mass awareness campaigns on antibiotic misuse through social media, television, mobile messaging, school curricula, and healthcare facilities	Lack of required resources and effectiveness of the dissemination			✓		✓	✓	✓	✓
	Engage NGOs and community or religious institutions in message dissemination and advocacy				✓		✓	✓	✓	✓
	Raise awareness that only registered physicians can prescribe antibiotics, and their prescribed courses must be completed				✓		✓	✓	✓	✓
**Barriers to quality healthcare access:**<br>▪ Inequitable and inadequate formal healthcare facilities.<br>▪ Reluctance to visit registered physicians due to financial constraints<br>▪ Insufficient counselling by physicians<br>▪ Empirical antibiotic prescription by physicians<br>▪ Absence of a digital database of patients and referral system	Extend service hours in public healthcare facilities.	Lack of Human resources for health	✓				✓	✓		✓
	Utilize mid- and lower-level healthcare providers for initial screenings and non-critical cases to reduce registered physicians' workload	Lack of government willingness to implement and understand the necessity, with regular monitoring requiring resources	✓						✓	
	Implement an effective referral system	Reforming the healthcare system is a difficult, time-consuming and resource-driven matter	✓						✓	
	Expand diagnostic facilities at public healthcare centres, referring uncommon tests to district-level public or affordable private labs via public-private partnerships	Require significant skilled human resources and financial resources.	✓				✓	✓	✓	✓

	To ensure patient safety and accountability, the government should invest in a patient database accessible to registered physicians for reviewing patient histories		✓						✓	
**Poor understanding on antibiotics**<br>▪ Limited understanding of the consequences of inappropriate antibiotic use<br>▪ People cannot distinguish between antibiotics and non-antibiotics, using them interchangeably<br>▪ Self-medications with antibiotics (SMA)	Registered physicians should counsel patients on appropriate use.	Difficult to manage time considering the volume of patients		✓			✓	✓	✓	✓
	Drug-sellers can play a key role in counselling customers on proper dosage	Pharmacy drug-sellers need training on antibiotic dispensing and effective counselling		✓			✓	✓	✓	✓
	Patient should ask healthcare providers or drug-sellers if any of the medications contain antibiotics	Limited awareness and understand the necessity of appropriate antibiotic use				✓	✓	✓	✓	✓
	When consulting healthcare providers or purchasing antibiotics, ask about dosage and duration, and buy the full course					✓	✓	✓	✓	✓
	Buy and complete the full course of antibiotics.	Inability to afford full antibiotic courses and lack of awareness about completing the course				✓	✓	✓	✓	✓
**Poor adherence to antibiotic regimens:**<br>▪ Discontinuing antibiotics once symptoms improve<br>▪ Low adherence to appropriate use of antibiotics	Antibiotic prices should be reduced and regulated by the government.	Oppositions from pharmaceutical companies and potential deterioration of drug quality	✓				✓			✓
	Ensure availability of antibiotics at all healthcare facilities	Lack of required resources and free antibiotics may cause misuse	✓				✓		✓	✓

▪ Availability of antibiotics without a full course at pharmacy	Pharmaceutical companies should package antibiotics with the minimum dosage	It may pressure customers with limited income, making it difficult to afford the full course at once.		✓			✓	✓	✓	
	Pharmacies should be prohibited from breaking antibiotic packages.			✓			✓	✓	✓	
	Pharmaceutical companies should use red-label antibiotic packaging in marketing to appropriate use, prescription requirements, and the consequences of misuse.	Changes in packaging requires time and huge costs		✓			✓	✓	✓	
**Approaches to clarify provider roles and standardize prescribing practices:**<br>▪ Non-uniform practice of proper antibiotic use regarding selection and dosage among providers<br>▪ People cannot differentiate qualified from unqualified healthcare providers.<br>▪ Unclear and undefined roles and restrictions for informal healthcare providers	The government should ensure registered physicians are aware of and adhere to antibiotic Standard Treatment Guidelines, while increasing monitoring and accountability among healthcare providers.	A clear health policy, resource-intensive digital monitoring system, and health system reform are required to address providers' long-standing empirical practices and ensure timely compliance.	✓						✓	
	Develop clear prescribing guidelines for non-MBBS providers and prohibit them from using the title "Dr."	Lack of government commitment and prioritization, with resistance from low- and mid-level healthcare providers.	✓					✓	✓	
**Commercial influence on prescribing:**<br>▪ Sales target in competitive pharmaceutical industries	Government should have a clear policy about antibiotic marketing strategy and ensure effective implementation	Lack of government commitment and prioritization, coupled with limited resources for monitoring.	✓				✓	✓	✓	

▪ Pharmaceuticals influence prescriptions by both formal and informal providers.<br>▪ Prioritizing corporate interests and downplaying misuse risks	Cancel registration of companies producing substandard drugs and enforce strict rules against unethical incentive-based marketing.		✓				✓	✓	✓	
	Use generic antibiotic names in prescriptions instead of brand names.	Using generic names may lead to misuse by pharmacy sellers and face opposition from leading pharmaceutical companies.	✓				✓			

Table 3: Co-designed AMS strategies & alignment with AMR-National Action Plan (NAP) [1]

Proposed AMS Strategy	Implementation Priority	Mentioned in NAP	Section	Page	Details from NAP
Formulate context-specific laws with monitoring and penalties	High	Directly	6.2.2	38	Strengthen regulatory frameworks and enforcement mechanisms for antimicrobial use and sales.
Monitor pharmacy dispensing and enforce penalties	High	Directly	6.2.2	38	Regular monitoring of antimicrobial dispensing and strict enforcement of prescription-only policies.
Mandatory short course for drug-sellers	High	Directly	6.2.3	39	Training and certification for drug-sellers as part of AMS capacity building.
Establish a complaint hotline	Moderate	Indirectly	6.2.4	40	Public engagement and feedback mechanisms mentioned, not specifically a hotline.
Engage district-level pharmacy associations	High	Indirectly	6.2.3	39	Collaboration with professional associations for AMS implementation.
Mass awareness campaigns via media, schools, healthcare	High	Directly	6.2.4	40	Awareness campaigns through media, education, and healthcare systems.
Engage NGOs and religious institutions	High	Indirectly	6.2.4	40	Community engagement encouraged, including civil society and religious groups.
Promote physician-only prescriptions	High	Directly	6.2.2	38	Prescription-only policy and adherence to treatment guidelines emphasized.
Extend service hours in public facilities	Moderate	Indirectly	6.2.1	37	Improving access to healthcare services through facility strengthening.
Use mid/lower-level providers for screening	Moderate	Indirectly	6.2.1	37	Task-shifting and capacity building for mid-level providers.
Implement referral system	High	Indirectly	6.2.1	37	Referral mechanisms mentioned under health system strengthening.
Expand diagnostics in public centers and refer rare tests	High	Directly	6.2.5	41	Improving diagnostic capacity and referral systems for specialized tests.
Create patient database for accountability	Moderate	Directly	6.2.5	41	Digital health records and data systems for tracking antimicrobial use.
Physician counselling on dosage and completion	High	Directly	6.2.2	38	Physician-led education and counselling on antimicrobial use.

Train drug-sellers for proper guidance	High	Directly	6.2.3	39	Training programs for drug-sellers on AMS practices.
Encourage patients to ask about antibiotics	High	Indirectly	6.2.4	40	Promoting patient awareness and engagement in treatment decisions.
Regulate and reduce antibiotic prices	Moderate	Indirectly	6.2.6	42	Affordability and access to quality antimicrobials addressed.
Ensure availability in facilities	Moderate	Directly	6.2.6	42	Ensuring availability of essential antimicrobials in public facilities.
Package full courses	Moderate	Indirectly	6.2.6	42	Packaging and labelling standards to promote full course adherence.
Ban breaking packs	Moderate	Indirectly	6.2.6	42	Discouraging partial dispensing and promoting complete treatment.
Use red-label packaging	Moderate	Indirectly	6.2.6	42	Visual cues and packaging to reinforce responsible use.
Enforce Standard Treatment Guidelines	Moderate	Directly	6.2.2	38	Mandatory adherence to national treatment protocols.
Develop guidelines for non-MBBS providers	Moderate	Indirectly	6.2.2	38	Guidance for informal and mid-level providers to ensure safe prescribing.
Regulate antibiotic marketing	Moderate	Directly	6.2.2	38	Oversight of pharmaceutical marketing practices.
Cancel licenses for substandard producers	Moderate	Directly	6.2.2	38	Regulatory actions against non-compliant manufacturers.
Promote generic prescriptions	Moderate	Indirectly	6.2.2	38	Encouraging use of generic names to reduce cost and improve access.
